# Supplementary material for: Using Serological Markers for the Surveillance of Plasmodium vivax Malaria: A Scoping Review
Source: Pathogens. 2023 May 31;12(6):791. doi: 10.3390/pathogens12060791 (PMC10302697; doi:10.3390/pathogens12060791)
Supplement: Supplementary file 1 [file pathogens-12-00791-s001.zip › pathogens-2381193-supplementary.pdf]

Table S1. - PRISMA Extension for Scoping Reviews (PRISMA-ScR): Checklist and Explanation.

SECTION	ITEM	PRISMA-ScR CHECKLIST ITEM	REPORTED ON PAGE #
		**TITLE**	
Title	1	Identify the report as a scoping review.	1
		**ABSTRACT**	
Structured summary	2	Provide a structured summary that includes (as applicable): background, objectives, eligibility criteria, sources of evidence, charting methods, results, and conclusions that relate to the review questions and objectives.	1
		**INTRODUCTION**	
Rationale	3	Describe the rationale for the review in the context of what is already known. Explain why the review questions/objectives lend themselves to a scoping review approach.	1-3
Objectives	4	Provide an explicit statement of the questions and objectives being addressed with reference to their key elements (e.g., population or participants, concepts, and context) or other relevant key elements used to conceptualize the review questions and/or objectives.	3
		**METHODS**	
Protocol and registration	5	Indicate whether a review protocol exists; state if and where it can be accessed (e.g., a Web address); and if available, provide registration information, including the registration number.	3
Eligibility criteria	6	Specify characteristics of the sources of evidence used as eligibility criteria (e.g., years considered, language, and publication status), and provide a rationale.	3
Information sources	7	Describe all information sources in the search (e.g., databases with dates of coverage and contact with authors to identify additional sources), as well as the date the most recent search was executed.	3
Search	8	Present the full electronic search strategy for at least 1 database, including any limits used, such that it could be repeated.	3
Selection of sources of evidence	9	State the process for selecting sources of evidence (i.e., screening and eligibility) included in the scoping review.	3
Data charting process	10	Describe the methods of charting data from the included sources of evidence (e.g., calibrated forms or forms that have been tested by the team before their use, and whether data charting was done independently or in duplicate) and any processes for obtaining and confirming data from investigators.	3-4
Data items	11	List and define all variables for which data were sought and any assumptions and simplifications made.	3-4
Critical appraisal of individual sources of evidence	12	If done, provide a rationale for conducting a critical appraisal of included sources of evidence; describe the methods used and how this information was used in any data synthesis (if appropriate).	N/A
Synthesis of results	13	Describe the methods of handling and summarizing the data that were charted.	3-4

**RESULTS**			
Selection of sources of evidence	14	Give numbers of sources of evidence screened, assessed for eligibility, and included in the review, with reasons for exclusions at each stage, ideally using a flow diagram.	4
Characteristics of sources of evidence	15	For each source of evidence, present characteristics for which data were charted and provide the citations.	4-5, S2
Critical appraisal within sources of evidence	16	If done, present data on critical appraisal of included sources of evidence (see item 12).	N/A
Results of individual sources of evidence	17	For each included source of evidence, present the relevant data that were charted that relate to the review questions and objectives.	5-8
Synthesis of results	18	Summarize and/or present the charting results as they relate to the review questions and objectives.	5-9
**DISCUSSION**			
Summary of evidence	19	Summarize the main results (including an overview of concepts, themes, and types of evidence available), link to the review questions and objectives, and consider the relevance to key groups.	11-12
Limitations	20	Discuss the limitations of the scoping review process.	12-13
Conclusions	21	Provide a general interpretation of the results with respect to the review questions and objectives, as well as potential implications and/or next steps.	13
**FUNDING**			
Funding	22	Describe sources of funding for the included sources of evidence, as well as sources of funding for the scoping review. Describe the role of the funders of the scoping review.	13

Adapted from: [27].

Table S2. – List of Included studies.

Title	Authors	Year
The hide and seek of Plasmodium vivax in West Africa: report from a large-scale study in Beninese asymptomatic subjects. [97]	Poirier P; Doderer-Lang C; Atchade PS; Lemoine JP; de l'Isle MC; Abou-Bacar A; Pfaff AW; Brunet J; Arnoux L; Haar E; Filisetti D; Perrotey S; Chabi NW; Akpovi CD; Anani L; Bigot A; Sanni A; Candolfi E	2016
Probability of antibody formation against circumsporozoite protein of Plasmodium vivax among Korean malaria patients. [57]	Nam HW; Song KJ; Ahn HJ; Yang Z; Chong CK; Cho PY; Ahn SK; Kim TS	2014
IgG isotype to C-terminal 19 kDa of Plasmodium vivax merozoite surface protein 1 among subjects with different levels of exposure to malaria in Brazil. [42]	Morais CG; Soares IS; Carvalho LH; Fontes CJ; Krettli AU; Braga EM	2005
Heterogeneous malaria transmission in long-term Afghan refugee populations: a cross-sectional study in five refugee camps in northern Pakistan. [119]	Wahid S; Stresman GH; Kamal SS; Sepulveda N; Kleinschmidt I; Bousema T; Drakeley C	2016
Seroprevalence of Plasmodium vivax Circumsporozoite Protein Antibody in High-Risk Malaria Areas in Korea. [126]	Lee J; Jin K; Ahn SK; Lee SK; Kwon HW; Na BK; Kim TS	2021
Epidemiological and ecological aspects related to malaria in the area of influence of the lake at Porto Primavera dam, in western São Paulo State, Brazil. [61]	Gomes Ade C; Paula MB; Duarte AM; Lima MA; Malafronte Rdos S; Mucci LF; Gotlieb SL; Natal D	2008
Seroprevalence of antibodies to repetitive domains of Plasmodium vivax circumsporozoite protein in United Arab Emirates children. [73]	Abu-Zeid YA; Alwash R; Shaheen HM; Bin-Othman SA; Lukic ML; Amiri KM; Charoenvit Y	2002
Malaria epidemiology in low-endemicity areas of the Atlantic Forest in the Vale do Ribeira, São Paulo, Brazil. [52]	Curado I; Dos Santos Malafronte R; de Castro Duarte AM; Kirchgatter K; Branquinho MS; Bianchi Galati EA	2006
Screening for malaria antigen and anti-malarial IgG antibody in forcibly-displaced Myanmar nationals: Cox's Bazar district, Bangladesh, 2018. [125]	Lu A; Cote O; Dimitrova SD; Cooley G; Alamgir A; Uzzaman MS; Flora MS; Widiati Y; Akhtar MS; Vandenent M; Ehlman DC; Bennett SD; Feldstein LR; Rogier E	2020
Seroprevalence of malaria infections in Korean troops on a peacekeeping mission in East Timor from 2001 to 2002. [70]	Chang EA; Park I; Kim JY; Suh IB; An SS; Lim CS; Kim YK; Lee KN	2004
Seroprevalence of Plasmodium vivax in the Republic of Korea (2003-2005) using indirect fluorescent antibody test. [68]	Kim TS; Kang YJ; Lee WJ; Na BK; Moon SU; Cha SH; Lee SK; Park YK; Pak JH; Cho PY; Sohn Y; Lee HW	2014
Naturally acquired antibodies to merozoite surface protein (MSP)-1(19) and cumulative exposure to Plasmodium falciparum and Plasmodium vivax in remote populations of the Amazon Basin of Brazil. [53]	Ladeia-Andrade S; Ferreira MU; Scopel KK; Braga EM; Bastos Mda S; Wunderlich G; Coura JR	2007
Plasmodium vivax and Plasmodium falciparum infections in the Republic of Djibouti: evaluation of their prevalence and potential determinants. [116]	Khaireh BA; Briolant S; Pascual A; Mokrane M; Machault V; Travaillé C; Khaireh MA; Farah IH; Ali HM; Abdi AI; Ayeh SN; Darar HY; Ollivier L; Waiss MK; Bogreau H; Rogier C; Pradines B	2012
Prevalence of Plasmodium vivax VK210 and VK247 subtype in Myanmar. [45]	Kim TS; Kim HH; Lee SS; Na BK; Lin K; Cho SH; Kang YJ; Kim DK; Sohn Y; Kim H; Lee HW	2010
Current and cumulative malaria infections in a setting embarking on elimination: Amhara, Ethiopia. [82]	Yalew WG; Pal S; Bansil P; Dabbs R; Tetteh K; Guinovart C; Kalnoky M; Serda BA; Tesfay BH; Beyene BB; Seneviratne C;	2017

	Littrell M; Yokobe L; Noland GS; Domingo GJ; Getachew A; Drakeley C; Steketee RW	
Spatial cluster analysis of Plasmodium vivax and P. malariae exposure using serological data among Haitian school children sampled between 2014 and 2016. [30]	Oviedo A; Herman C; Knipes A; Worrell CM; Fox LM; Desir L; Fayette C; Javel A; Monestime F; Mace KE; Chang MA; Lemoine JF; Won K; Udhayakumar V; Rogier E	2022
Multiplex serology demonstrate cumulative prevalence and spatial distribution of malaria in Ethiopia. [85]	Assefa A; Ali Ahmed A; Deressa W; Sime H; Mohammed H; Kebede A; Solomon H; Teka H; Gurrala K; Matei B; Wakeman B; Wilson GG; Sinha I; Maude RJ; Ashton R; Cook J; Shi YP; Drakeley C; von Seidlein L; Rogier E; Hwang J	2019
Sequence conservation of Plasmodium vivax glutamate dehydrogenase among Korean isolates and its application in seroepidemiology. [122]	Seol B; Shin HI; Kim JY; Jeon BY; Kang YJ; Pak JH; Kim TS; Lee HW	2017
Serological measures to assess the efficacy of malaria control programme on Ambae Island, Vanuatu. [88]	Idris ZM; Chan CW; Mohammed M; Kalkoa M; Taleo G; Junker K; Arcà B; Drakeley C; Kaneko A	2017
Malaria epidemiology in low-endemicity areas of the northern coast of Ecuador: high prevalence of asymptomatic infections. [7]	Sáenz FE; Arévalo-Cortés A; Valenzuela G; Vallejo AF; Castellanos A; Poveda-Loayza AC; Gutierrez JB; Alvarez A; Yan YH; Benavides Y; Castro LE; Arévalo-Herrera M; Herrera S	2017
Serologically defined variations in malaria endemicity in Pará state, Brazil. [118]	Cunha MG; Silva ES; Sepúlveda N; Costa SP; Saboia TC; Guerreiro JF; Póvoa MM; Corran PH; Riley E; Drakeley CJ	2014
Malaria serology data from the Guiana shield: first insight in IgG antibody responses to Plasmodium falciparum, Plasmodium vivax and Plasmodium malariae antigens in Suriname. [124]	Labadie-Bracho MY; van Genderen FT; Adhin MR	2020
A multianalyte Dot-ELISA for simultaneous detection of malaria, Chagas disease, and syphilis-specific IgG antibodies. [33]	Coelho JS; Soares IS; Lemos EA; Jimenez MC; Kudó ME; Moraes SL; Ferreira AW; Sanchez MC	2007
Malaria epidemiology in central Myanmar: identification of a multi-species asymptomatic reservoir of infection. [121]	Ghinai I; Cook J; Hla TT; Htet HM; Hall T; Lubis IN; Ghinai R; Hesketh T; Naung Y; Lwin MM; Latt TS; Heymann DL; Sutherland CJ; Drakeley C; Field N	2017
Prevalence of antibodies to the circumsporozite protein of Plasmodium vivax in five different regions of Korea. [101]	Lee KN; Suh IB; Chang EA; Kim SD; Cho NS; Park PW; An SS; Park O; Lim C	2003
Specificity of the IgG antibody response to Plasmodium falciparum, Plasmodium vivax, Plasmodium malariae, and Plasmodium ovale MSP1(19) subunit proteins in multiplexed serologic assays. [37]	Priest JW; Plucinski MM; Huber CS; Rogier E; Mao B; Gregory CJ; Candrinho B; Colborn J; Barnwell JW	2018
Hotspots of Malaria Transmission in the Peruvian Amazon: Rapid Assessment through a Parasitological and Serological Survey. [75]	Rosas-Aguirre A; Speybroeck N; Llanos-Cuentas A; Rosanas-Urgell A; Carrasco-Escobar G; Rodriguez H; Gamboa D; Contreras-Mancilla J; Alava F; Soares IS; Remarque E; D Alessandro U; Erhart A	2015
Seroprevalence to the circumsporozoite protein peptide antigen of Plasmodium vivax in Korean children. [48]	Lim CS; Yoon JK; Chang EA; Suh IB; An SS; Lee KH; Chung JT; Tockgo YC	2005
Sero-epidemiological evaluation of changes in Plasmodium falciparum and Plasmodium vivax	Cook J; Speybroeck N; Sochanta T; Somony H; Sokny M; Claes F; Lemmens K; Theisen	2012

transmission patterns over the rainy season in Cambodia. [81]	M; Soares IS; D'Alessandro U; Coosemans M; Erhart A	
Detection of antibodies against the CB9 to ICB10 region of merozoite surface protein-1 of Plasmodium vivax among the inhabitants in epidemic areas. [77]	Kim TS; Sohn Y; Kim JY; Lee WJ; Na BK; Kang YJ; Lee HW	2014
Prevalence and level of antibodies to the circumsporozoite protein of human malaria parasites in five states of the Amazon region of Brazil. [54]	Arruda ME; Zimmerman RH; Souza RM; Oliveira-Ferreira J	2007
Molecular cloning and expression of the VK247 circumsporozoite protein for serodiagnosis of variant form Plasmodium vivax. [35]	Kim TS; Kim HH; Lee SS; Oh CM; Choi KM; Lin K; Kim JY; Na BK; Han ET; Sohn Y; Kim H; Lee HW	2011
Evaluation of Plasmodium vivax ELISA for the blood screen. [40]	Nam MH; Kim JS; Cho CH; Han ET; Lee WJ; Lee HK; An SS; Lim CS; Lee KN	2010
Serologic responses of Korean soldiers serving in malaria-endemic areas during a recent outbreak of Plasmodium vivax. [72]	Park CG; Chwae YJ; Kim JI; Lee JH; Hur GM; Jeon BH; Koh JS; Han JH; Lee SJ; Park JW; Kaslow DC; Strickman D; Roh CS	2000
Characterization of Plasmodium vivax heat shock protein 70 and evaluation of its value for serodiagnosis of tertian malaria. [36]	Na BK; Park JW; Lee HW; Lin K; Kim SH; Bae YA; Sohn WM; Kim TS; Kong Y	2007
Efficacy of the merozoite surface protein 1 of Plasmodium vivax as an antigen for ELISA to diagnose malaria. [32]	Kim YM; Hwang HA; Yun WS; Kim SI; Lee KW; Park SK; Lee YJ; Kim TK; Wongsrichanalai C; Sakanari JA; Park H	2004
Detection of an antibody against Plasmodium vivax in residents of Gimpo-si, South Korea, using an indirect fluorescent antibody test. [114]	Lee WJ; Kim HH; Hwang SM; Park MY; Kim NR; Cho SH; In TS; Kim JY; Sattabongkot J; Sohn Y; Kim H; Lee JK; Lee HW	2011
Development and validation of serological markers for detecting recent Plasmodium vivax infection. [41]	Longley RJ; White MT; Takashima E; Brewster J; Morita M; Harbers M; Obadia T; Robinson LJ; Matsuura F; Liu ZSJ; Li-Wai-Suen CSN; Tham WH; Healer J; Huon C; Chitnis CE; Nguitragool W; Monteiro W; Proietti C; Doolan DL; Siqueira AM; Ding XC; Gonzalez IJ; Kazura J; Lacerda M; Sattabongkot J; Tsuboi T; Mueller I	2020
Serological evidence for a decline in malaria transmission following major scale-up of control efforts in a setting selected for Plasmodium vivax and Plasmodium falciparum malaria elimination in Babile district, Oromia, Ethiopia. [84]	Keffale M; Shumie G; Behaksra SW; Chali W; Hoogen LLVD; Hailemeskel E; Mekonnen D; Chanyalew M; Damte D; Fanta T; Ashine T; Chali S; Tetteh KKA; Birhanu DD; Balcha TT; Aseffa A; Drakeley C; Tessema TS; Adamu H; Bousema T; Gadisa E; Tadesse FG	2019
ELISA detection of vivax malaria with recombinant multiple stage-specific antigens and its application to survey of residents in endemic areas. [90]	Kim S; Ahn HJ; Kim TS; Nam HW	2003
Evaluation of Immunoglobulin G Responses to Plasmodium falciparum and Plasmodium vivax in Malian School Children Using Multiplex Bead Assay. [67]	Rogier E; Moss DM; Chard AN; Trinies V; Doumbia S; Freeman MC; Lammie PJ	2017
The shape of the iceberg: quantification of submicroscopic Plasmodium falciparum and Plasmodium vivax parasitaemia and gametocytaemia in five low endemic settings in Ethiopia. [94]	Tadesse FG; van den Hoogen L; Lanke K; Schildkraut J; Tetteh K; Aseffa A; Mamo H; Sauerwein R; Felger I; Drakeley C; Gadissa E; Bousema T	2017

Survey for asymptomatic malaria cases in low transmission settings of Iran under elimination programme. [55]	Zoghi S; Mehrizi AA; Raeisi A; Haghdoost AA; Turki H; Safari R; Kahanali AA; Zakeri S	2012
Evidence for the transmission of Plasmodium vivax in the Republic of the Congo, West Central Africa. [87]	Culleton R; Ndounga M; Zeyrek FY; Coban C; Casimiro PN; Takeo S; Tsuboi T; Yadava A; Carter R; Tanabe K	2009
Evaluation of circumsporozoite protein of Plasmodium vivax to estimate its prevalence in the Republic of Korea: an observational study of incidence. [66]	Cho PY; Lee SW; Ahn SK; Kim JS; Cha SH; Na BK; Park YK; Lee SK; Lee WJ; Nam HW; Hong SJ; Pak JH; Kang YJ; Sohn Y; Bahk YY; Cho HI; Kim TS; Lee HW	2013
Heterogeneity in response to serological exposure markers of recent Plasmodium vivax infections in contrasting epidemiological contexts. [23]	Rosado J; White MT; Longley RJ; Lacerda M; Monteiro W; Brewster J; Sattabongkot J; Guzman-Guzman M; Llanos-Cuentas A; Vinetz JM; Gamboa D; Mueller I	2021
Sero-identification of the aetiologies of human malaria exposure (Plasmodium spp.) in the Limu Kossa District of Jimma Zone, South western Ethiopia. [59]	Feleke SM; Brhane BG; Mamo H; Assefa A; Woyessa A; Ogawa GM; Cama V	2019
Molecular typing reveals substantial Plasmodium vivax infection in asymptomatic adults in a rural area of Cameroon. [71]	Fru-Cho J; Bumah VV; Safeukui I; Nkuo-Akenji T; Titanji VP; Haldar K	2014
Unexpected high circulation of Plasmodium vivax in asymptomatic children from Kédougou, southeastern Senegal. [92]	Niang M; Diop F; Niang O; Sadio BD; Sow A; Faye O; Diallo M; Sall AA; Perraut R; Toure-Balde A	2017
Using Respondent Driven Sampling to Identify Malaria Risks and Occupational Networks among Migrant Workers in Ranong, Thailand. [120]	Wangroongsarb P; Hwang J; Thwing J; Karuchit S; Kumpetch S; Rand A; Drakeley C; MacArthur JR; Kachur SP; Satimai W; Meek S; Sintasath DM	2016
IgG Antibody Responses Are Preferential Compared With IgM for Use as Serological Markers for Detecting Recent Exposure to Plasmodium vivax Infection. [43]	Longley RJ; White MT; Brewster J; Liu ZSJ; Bourke C; Takashima E; Harbers M; Tham WH; Healer J; Chitnis CE; Monteiro W; Lacerda M; Sattabongkot J; Tsuboi T; Mueller I	2021
Estimating the malaria transmission of Plasmodium vivax based on serodiagnosis. [117]	Kim JY; Kim HH; Na BK; Kim YJ; Sohn Y; Kim H; Kim TS; Lee HW	2012
Cross sectional study reveals a high percentage of asymptomatic Plasmodium vivax infection in the Amazon Rio Negro area, Brazil. [113]	Suárez-Mutis MC; Cuervo P; Leoratti FM; Moraes-Avila SL; Ferreira AW; Fernandes O; Coura JR	2007
Serologic markers for detecting malaria in areas of low endemicity, Somalia, 2008. [65]	Bousema T; Youssef RM; Cook J; Cox J; Alegana VA; Amran J; Noor AM; Snow RW; Drakeley C	2010
Asymptomatic Plasmodium infection in a residual malaria transmission area in the Atlantic Forest region: Implications for elimination. [123]	Miguel RB; Albuquerque HG; Sanchez MCA; Coura JR; Santos SDS; Silva SD; Moreira CJC; Suárez-Mutis MC	2019
Geographical patterns of malaria transmission based on serological markers for falciparum and vivax malaria in Ratanakiri, Cambodia. [47]	Kerkhof K; Sluydts V; Heng S; Kim S; Pareyn M; Willen L; Canier L; Sovannaroth S; Ménard D; Sochantha T; Coosemans M; Durnez L	2016
Natural infections with different Plasmodium species induce antibodies reactive to a chimeric Plasmodium vivax recombinant protein. [29]	McCaffery JN; Singh B; Nace D; Moreno A; Udhayakumar V; Rogier E	2021
Detection of human malaria using recombinant Plasmodium knowlesi merozoire surface protein-1 ($MSP-1_{19}$) expressed in Escherichia coli. [34]	Sonaimuthu P; Cheong FW; Chin LC; Mahmud R; Fong MY; Lau YL	2015

Epidemiologic aspects of the malaria transmission cycle in an area of very low incidence in Brazil. [95]	Cerutti C Jr; Boulos M; Coutinho AF; Hatab Mdo C; Falqueto A; Rezende HR; Duarte AM; Collins W; Malafronte RS	2007
Malaria in pregnant women living in areas of low transmission on the southeast Brazilian Coast: molecular diagnosis and humoural immunity profile. [64]	Hristov AD; Sanchez MC; Ferreira JJ; Lima GF; Inoue J; Costa-Nascimento Mde J; Sanchez AR; Ramos-Sanchez EM; Di Santi SM	2014
Micro-epidemiology of malaria in an elimination setting in Central Vietnam. [46]	Bannister-Tyrrell M; Xa NX; Kattenberg JH; Van Van N; Dung VKA; Hieu TM; Van Hong N; Rovira-Vallbona E; Thao NT; Duong TT; Rosanas-Urgell A; Peeters Grietens K; Erhart A	2018
Assessment of antibody responses in local and immigrant residents of areas with autochthonous malaria transmission in Greece. [50]	Piperaki ET; Mavrouli M; Tseroni M; Routsias J; Kallimani A; Veneti L; Georgitsou M; Chania M; Georgakopoulou T; Hadjichristodoulou C; Tsakris A	2015
Anti-malarial seroprevalence assessment during an elimination programme in Chabahar District, south-eastern Iran. [79]	Zakeri S; van den Hoogen LL; Mehrizi AA; Karimi F; Raeisi A; Drakeley C	2016
Effectiveness of a serological tool to predict malaria transmission intensity in an elimination setting. [80]	Dewasurendra RL; Dias JN; Sepulveda N; Gunawardena GS; Chandrasekharan N; Drakeley C; Karunaweera ND	2017
Relationship between Antibody-Positive Rate against Plasmodium vivax Circumsporozoite Protein and Incidence of Malaria. [102]	Lee HW; Kang YJ; Cho SH; Na BK; Pak JH; Nam HW; Park YK; Sohn Y; Kim TS	2015
Analysis of anti-Plasmodium IgG profiles among Fulani nomadic pastoralists in northern Senegal to assess malaria exposure. [58]	Seck MC; Thwing J; Badiane AS; Rogier E; Fall FB; Ndiaye PI; Diongue K; Mbow M; Ndiaye M; Diallo MA; Gomis JF; Mbaye A; Ndiaye T; Gaye A; Sy M; Déme AB; Ndiaye YD; Ndiaye D	2020
Spatial Distribution of Plasmodium falciparum and Plasmodium vivax in Northern Ethiopia by Microscopic, Rapid Diagnostic Test, Laboratory Antibody, and Antigen Data. [100]	Leonard CM; Assefa A; Sime H; Mohammed H; Kebede A; Solomon H; Drakeley C; Murphy M; Hwang J; Rogier E	2022
Assessing malaria transmission in a low endemicity area of north-western Peru. [78]	Rosas-Aguirre A; Llanos-Cuentas A; Speybroeck N; Cook J; Contreras-Mancilla J; Soto V; Gamboa D; Pozo E; Ponce OJ; Pereira MO; Soares IS; Theisen M; D'Alessandro U; Erhart A	2013
Evaluating seroprevalence to circumsporozoite protein to estimate exposure to three species of Plasmodium in the Brazilian Amazon. [74]	Pereira VA; S√°nchez-Arcila JC; Vasconcelos MPA; Ferreira AR; de Souza Videira L; Teva A; Perce-da-Silva D; Marques MTQ; de Carvalho LH; Banic DM; Pôrto LCS; Oliveira-Ferreira J	2018
An immunomics approach for the analysis of natural antibody responses to Plasmodium vivax infection. [31]	Chen JH; Chen SB; Wang Y; Ju C; Zhang T; Xu B; Shen HM; Mo XJ; Molina DM; Eng M; Liang X; Gardner MJ; Wang R; Hu W	2015
Common asymptomatic and submicroscopic malaria infections in Western Thailand revealed in longitudinal molecular and serological studies: a challenge to malaria elimination. [8]	Baum E; Sattabongkot J; Sirichaisinthop J; Kiattibutr K; Jain A; Taghavian O; Lee MC; Huw Davies D; Cui L; Felgner PL; Yan G	2016
Characterization of Plasmodium falciparum and Plasmodium vivax recent exposure in an area of significantly decreased transmission intensity in Central Vietnam. [63]	Kattenberg JH; Erhart A; Truong MH; Rovira-Vallbona E; Vu KAD; Nguyen THN; Nguyen VH; Nguyen VV; Bannister-Tyrrell M; Theisen M; Bennet A; Lover AA; Tran TD; Nguyen XX; Rosanas-Urgell A	2018
Antibody profiles to plasmodium merozoite surface protein-1 in Cambodian adults during	Spring MD; Pichyangkul S; Lon C; Gosi P; Yongvanichit K; Srichairatanakul U;	2016

an active surveillance cohort with nested treatment study. [89]	Limsalakpeth A; Chaisatit C; Chann S; Sriwichai S; Auayapon M; Chaorattanakawee S; Dutta S; Prom S; Meng Chour C; Walsh DS; Angov E; Saunders DL	
Estimation on local transmission of malaria by serological approach under low transmission setting in Myanmar. [69]	Nyunt MH; Soe TN; Shein T; Zaw NN; Han SS; Muh F; Lee SK; Han JH; Park JH; Ha KS; Park WS; Hong SH; Kyaw MP; Han ET	2018
Implementation and application of a multiplex assay to detect malaria-specific antibodies: a promising tool for assessing malaria transmission in Southeast Asian pre-elimination areas. [39]	Kerkhof K; Canier L; Kim S; Heng S; Sochantha T; Sovannaroth S; Vigan-Womas I; Coosemans M; Sluydts V; Ménard D; Durnez L	2015
A sero-epidemiological study of malaria in human and monkey populations in French Guiana. [49]	Volney B; Pouliquen JF; De Thoisy B; Fandeur T	2002
Malaria in pregnancy: a passive surveillance study of pregnant women in low transmission areas of Colombia, Latin America.[62]	Lopez-Perez M; Pacheco MA; Buriticá L; Escalante AA; Herrera S; Arévalo-Herrera M	2016
Application of 23 Novel Serological Markers for Identifying Recent Exposure to Plasmodium vivax Parasites in an Endemic Population of Western Thailand. [9]	Chotirat S; Nekkab N; Kumpitak C; Hietanen J; White MT; Kiattibutr K; Sa-Angchai P; Brewster J; Schoffer K; Takashima E; Tsuboi T; Harbers M; Chitnis CE; Healer J; Tham WH; Nguitragool W; Mueller I; Sattabongkot J; Longley RJ	2021
Cross-sectional study of asymptomatic malaria and seroepidemiological surveillance of seven districts in Gia Lai province, Vietnam. [127]	San NN; Kien NX; Manh ND; Van Thanh N; Chavchich M; Binh NTH; Long TK; Edgel KA; Rovira-Vallbona E; Edstein MD; Martin NJ	2022
Serological markers to measure recent changes in malaria at population level in Cambodia. [38]	Kerkhof K; Sluydts V; Willen L; Kim S; Canier L; Heng S; Tsuboi T; Sochantha T; Sovannaroth S; Ménard D; Coosemans M; Durnez L	2016
Analysis of serological data to investigate heterogeneity of malaria transmission: a community-based cross-sectional study in an area conducting elimination in Indonesia. [103]	Surendra H; Wijayanti MA; Murhandarwati EH; Irnawati; Yuniarti T; Mardiati; Herdiana; Sumiwi ME; Hawley WA; Lobo NF; Cook J; Drakeley C; Supargiyono	2019
Prevalence and seroprevalence of Plasmodium infection in Myanmar reveals highly heterogeneous transmission and a large hidden reservoir of infection. [105]	Edwards HM; Dixon R; Zegers de Beyl C; Celhay O; Rahman M; Myint Oo M; Lwin T; Lin Z; San T; Thwe Han K; Myaing Nyunt M; Plowe C; Stresman G; Hall T; Drakeley C; Hamade P; Aryal S; Roca-Feltrer A; Hlaing T; Thi A	2021
Serologic markers in relation to parasite exposure history help to estimate transmission dynamics of Plasmodium vivax. [115]	Yildiz Zeyrek F; Palacpac N; Yuksel F; Yagi M; Honjo K; Fujita Y; Arisue N; Takeo S; Tanabe K; Horii T; Tsuboi T; Ishii KJ; Coban C	2011
Community-based molecular and serological surveillance of subclinical malaria in Myanmar. [83]	O'Flaherty K; Oo WH; Zaloumis SG; Cutts JC; Aung KZ; Thein MM; Drew DR; Razook Z; Barry AE; Parischa N; Zaw NN; Thu HK; Thi A; Htay WYM; Soe AP; Simpson JA; Beeson JG; Agius PA; Fowkes FJI	2021
Malaria transmission and individual variability of the naturally acquired IgG antibody against the Plasmodium vivax blood-stage antigen in an endemic area in Brazil. [51]	Costa EMF; Amador ECC; Silva ES; Alvarenga CO; Pereira PE; Póvoa MM; Cunha MG	2020

Using serological measures to monitor changes in malaria transmission in Vanuatu. [91]	Cook J; Reid H; Iavro J; Kuwahata M; Taleo G; Clements A; McCarthy J; Vallely A; Drakeley C	2010
Geostatistical modeling of malaria endemicity using serological indicators of exposure collected through school surveys. [112]	Ashton RA; Kefyalew T; Rand A; Sime H; Assefa A; Mekasha A; Edosa W; Tesfaye G; Cano J; Teka H; Reithinger R; Pullan RL; Drakeley CJ; Brooker SJ	2015
Integration of Multiplex Bead Assays for Parasitic Diseases into a National, Population-Based Serosurvey of Women 15-39 Years of Age in Cambodia. [86]	Priest JW; Jenks MH; Moss DM; Mao B; Buth S; Wannemuehler K; Soeung SC; Lucchi NW; Udhayakumar V; Gregory CJ; Huy R; Muth S; Lammie PJ	2016
Antibody Profile Comparison against MSP1 Antigens of Multiple Plasmodium Species in Human Serum Samples from Two Different Brazilian Populations Using a Multiplex Serological Assay. [60]	Monteiro EF; Fernandez-Becerra C; Curado I; Wunderlich G; Hiyane MI; Kirchgatter K	2021
Submicroscopic and asymptomatic Plasmodium falciparum and Plasmodium vivax infections are common in western Thailand - molecular and serological evidence. [6]	Baum E; Sattabongkot J; Sirichaisinthop J; Kiattibutr K; Davies DH; Jain A; Lo E; Lee MC; Randall AZ; Molina DM; Liang X; Cui L; Felgner PL; Yan G	2015
Blood-stage Plasmodium vivax antibody dynamics in a low transmission setting: A nine year follow-up study in the Amazon region. [44]	Pires CV; Alves JRS; Lima BAS; Paula RB; Costa HL; Torres LM; Sousa TN; Soares IS; Sanchez BAM; Fontes CJF; Ntumngia FB; Adams JH; Kano FS; Carvalho LH	2018
Using health facility-based serological surveillance to predict receptive areas at risk of malaria outbreaks in elimination areas. [76]	Surendra H; Supargiyono; Ahmad RA; Kusumasari RA; Rahayujati TB; Damayanti SY; Tetteh KKA; Chitnis C; Stresman G; Cook J; Drakeley C	2020
Surveillance of Plasmodium vivax transmission using serological models in the border areas of China-Myanmar. [15]	Yao M; Xiao L; Sun X; Lin Z; Hao X; Bai QQ; Yin DH	2022
Surveillance on the Vivax Malaria in Endemic Areas in the Republic of Korea Based on Molecular and Serological Analyses. [93]	Lee SK; Hu F; Firdaus ER; Park JH; Han JH; Lee SE; Shin HI; Cho SH; Park WS; Lu F; Han ET	2020

Table S3. – Characteristics of Included Studies.

Study ID	Region	Study Design	Total no. participants	No. antigens	Antibody type
Kim, 2004 [32]	Thailand, Korea	CS	306	1	IgG, IgM
Coelho, 2007 [33]	Brazil	VS	103	1	IgG
Sonaimuthu, 2015 [34]	DNS	VS	215	1	IgG
Kim, 2011 [35]	Myanmar	CS	100	1	IgG
Na, 2007 [36]	Korea, Myanmar	CS	278	1	IgG
McCaffery, 2021 [29]	United States	CS	239	2	IgG
Rogier, 2017 [67]	Mali	CS	805	1	IgG
Priest, 2018 [37]	Brazil	Specificity study	N/A	1	IgG
Seol, 2017 [122]	Korea	CS	100	1	IgG
Kattenberg, 2018 [63]	Vietnam	CH	429	2	IgG
Chang, 2004 [70]	East Timor	CS	210	2	IgG
Lee, 2003 [101]	Korea	CS	1014	1	IgG
Lim, 2005 [48]	Korea	CS	1176	1	IgG
Morais, 2005 [42]	Brazil	CS	354	1	IgG
Park, 2000 [72]	Korea	CS	1713	3	IgG, IgM
Kim, 2014a [68]	Korea	CS	1774	1	IgG
Hristov, 2014 [64]	Brazil	CH	125	1	IgG
Abu-Zeid, 2002 [73]	United Arab Emirates	CS	1006	3	IgG
Cho, 2013 [66]	Korea	CS	N/A	1	N/A
Lee, 2011 [114]	Korea	CS	5797	1	IgG
Yildiz Zeyrek, 2011 [115]	Turkey	CS	195	4	IgG, IgM
Volney, 2002 [49]	Guinea	CS	218	3	IgG
Piperaki, 2015 [50]	Greece	CS	1057	2	IgG, IgM
Kerkhof, 2015 [39]	Cambodia	CS	2000	7	IgG
Nam, 2014 [57]	Korea, India, Ugana	VS	126	2	N/A
Kerkhof, 2016b [38]	Cambodia	CS	8654	7	IgG
Priest, 2016 [86]	Cambodia	CS	2154	1	IgG
Bousema, 2010 [65]	Somalia	CS	1044	2	IgG
Kim, 2014b [77]	Korea	CS	1774	1	IgG
Lopez-Perez, 2016 [62]	Colombia	DC	34	2	IgG
Kim, 2012 [117]	Korea	CS	1547	N/A	IgG
Chen, 2015 [31]	China	CS	25	N/A	IgG
Nam, 2010 [40]	Korea	CS	911	2	IgG, IgM
Arruda, 2007 [54]	Brazil	CS	1206	2	N/A
Gomes, 2008 [61]	Brazil	CS	216	3	IgG
Ladeia-Andrade, 2007 [53]	Brazil	CS	473	1	IgG
Curado, 2006 [52]	Brazil	CS	318	3	IgG, IgM
Fru-Cho, 2014 [71]	Cameroon	CS	269	2	N/A
Ashton, 2015 [112]	Ethiopia	CS	6609	2	IgG
Wahid, 2016 [119]	Pakistan	CS	2522	2	N/A
Cerruti Junior, 2007 [95]	Brazil	CS	1842	N/A	IgG, IgM
Zakeri, 2016 [79]	Iran	CS	1479	2	x

Zoghi, 2012 [55]	Iran	CS	1000	1	IgG
Cook, 2010 [91]	Vanuatu	CS	1766	2	IgG
Seck, 2020 [58]	Senegal	CS	1472	1	IgG
Dewasurendra, 2017 [80]	Sri Lanka	CS	1186	2	IgG
Rosas-Aguirre, 2013 [78]	Peru	CS	2267	1	IgG
Pereira, 2018 [74]	Brazil	CS	357	1	IgG
Leonard, 2022 [100]	Ethiopia	CS	2279	3	IgG
Lee, 2015 [102]	Korea	CS	1774	1	IgG
Bannister-Tyrrell, 2018 [46]	Vietnam	CS	186	2	N/A
Lee, 2021 [126]	Korea	CS	3477	1	IgG
Cook, 2012 [81]	Cambodia	CS	804	1	IgG
Costa, 2020 [51]	Brazil	CS	462	1	IgG
Baum, 2015 [6]	Thailand	CS	280	515	N/A
O'Flaherty, 2021 [83]	Myanmar	RCT	10857	1	IgG
Suárez-Mutis, 2007 [113]	Brazil	CS	109	1	IgG
Spring, 2016 [89]	Cambodia	CH	222	1	IgG
Culleton, 2009 [87]	Republic of the Congo	CS	409	2	IgG
Baum, 2016 [8]	Thailand	CS	298	515	N/A
Wangroongsarb, 2016 [120]	Thailand	CS	1242	4	N/A
Kim, 2003 [90]	Korea	CS	3262	5	IgG
Idris, 2017 [88]	Vanuatu	CS	513	3	IgG
Miguel, 2019 [123]	Brazil	CS	314	1	IgG
Rosas-Aguirre, 2015 [75]	Brazil	CS	651	2	IgG
Lu, 2020 [125]	Bangladesh	CS	1239	1	IgG
Longley, 2021 [43]	Thailand, Brazil	CH	1754	20	IgG, IgM
Nyunt, 2018 [69]	Myanmar	CH	1182	3	IgG
Chotirat, 2021 [9]	Thailand	CS	4255	23	IgG
Sáenz, 2017 [7]	Ecuador	CS	648	2	IgG
Cunha, 2014 [118]	Brazil	CS	1330	3	IgG
Ghinai, 2017 [121]	Myanmar	CS	1638	1	IgG
Surendra, 2019 [103]	Indonesia	CS	1624	2	IgG
Feleke, 2019 [59]	Ethiopia	CS	180	1	IgG
Rosado, 2021 [23]	Peru	VS	590	34	IgG
Pires, 2018 [44]	Brazil	CH	102	4	IgG
Niang, 2017 [92]	Senegal	Retrospective Serological Study	48	1	IgG
Monteiro, 2021 [60]	Brazil	Retrospective Serological Study	416	1	IgG
Yalew, 2017 [82]	Ethiopia	CS	7878	2	IgG
Surendra, 2020 [76]	Indonesia	CS	9453	5	N/A
Oviedo, 2022 [30]	Haiti	CS	24,559	1	IgG
Kerkhof, 2016a [47]	Cambodia	CS	6502	2	IgG
Edwards, 2021 [105]	Myanmar	CS	11,653	2	IgG
San, 2022 [127]	Vietnam	CS	3283	4	IgG

Yao, 2022 [15]	China	CS	3064	3	N/A
Keffale, 2019 [84]	Ethiopia	CS	1144	1	IgG
Kim, 2010 [45]	Myanmar	CS	100	1	IgG
Tadesse, 2017 [94]	Ethiopia	CS	845	2	N/A
Poirier, 2016 [97]	Benin	CS	1234	2	IgG
Khaireh, 2012 [116]	Djibouti	CS	1910	2	IgG
Labadie-Bracho, 2020 [124]	Suriname	CS	197	1	IgG
Lee, 2020 [93]	Korea	CS	777	3	IgG
Assefa, 2019 [85]	Ethiopia	CS	53,335	2	IgG
Longley, 2020 [41]	Thailand, Brazil	CH	65	342	IgG

CS = cross-sectional, CH = cohort, VS = validation study, DC = descriptive study, N/A = not available.
